# Goblet Cell Carcinoid in a Patient with Neurofibromatosis Type 1: A Rare Combination

**DOI:** 10.1155/2012/185730

**Published:** 2012-12-04

**Authors:** Tine Gregersen, Nanna Holt, Henning Gronbaek, Ida Vogel, Lars J. Jørgensen, Klaus Krogh

**Affiliations:** ^1^Department of Medicine, Hepatology and Gastroenterology, Aarhus University Hospital, Norrebrogade 44, 8000 Aarhus C, Denmark; ^2^Department of Clinical Genetics, Aarhus University Hospital, Brendstrupgaardsvej 21 C, 8200 Aarhus N, Denmark; ^3^Institute of Pathology, Vendsyssel Hospital, Bispensgade 37, 9800 Hjorring, Denmark

## Abstract

Neuroendocrine tumors are rare tumors primarily located in the gastrointestinal tract. Goblet cell carcinoid is a rare subgroup of neuroendocrine tumors located in the appendix. Neurofibromatosis type 1 is an autosomal dominant disorder caused by a mutation in the *NF1* gene. Patients with neurofibromatosis type 1 have an increased incidence of typical neuroendocrine tumors, but it is unknown if this is the case with goblet cell carcinoids. We describe a patient with both neurofibromatosis type 1 and goblet cell carcinoid, that according to literature would occur in 0.00017 per million per year. This may suggest a previously unknown association between neurofibromatosis type 1 and goblet cell carcinoids.

## 1. Introduction

Neuroendocrine tumors (NETs) are rare with an estimated prevalence of 35/100.000 per year [1]. The most frequent location of NETs is the gastrointestinal tract where most originate from enterochromaffin (EC) cells [2, 3]. These cells originate from the neural crest and constitute the diffuse neuroendocrine system. Goblet cell carcinoid (GCC) tumors are a very rare subgroup of mucin producing NETs usually arising in the appendix. The incidence of GCCs is approximately 0.5 per million/year [4]. In contrast to other NETs, GCCs have a mixed phenotype with partial neuroendocrine differentiation and partial intestinal type goblet cell morphology [5]. 

Neurofibromatosis type 1 (NF1) is an autosomal dominant inherited disorder with an estimated prevalence of 1/3.000 [6]. NF1 is characterised by multiple café au lait spots, axillary and inguinal freckling, cutaneous neurofibromas, and hamartomas of the iris. Learning disabilities are seen in more than half of the affected individuals. Less common, but potentially more harmful manifestations are plexiform neurofibromas, optic tumors, gliomas in the central nervous system, vasculopathy, and an increased risk of malignancies. The disease is caused by heterozygote loss-of-function mutations in the *NF1* gene, and more than half of all patients have de novo germ line mutations. For unknown reasons the mutation rate for the *NF1* gene is among the highest observed. The function of the protein neurofibromin is not fully known, but it is a multidomain molecule with the capacity to regulate several intracellular processes such as RAS-cyclic AMP and ERK/MAP kinase cascades. Thus, it functions as a tumor suppressor gene. The development of tumors, café au lait spots, and neurofibromas is caused by spontaneous somatic mutations in the one remaining intact *NF1* gene leaving these cells unable to express neurofibromin. Thereby, the disease becomes progressive throughout life, and the rate of spontaneous somatic mutation determines the severity of disease manifestations as well as the risk of cancers in tissues of neuroectodermal origin. Consequently, NF1 is associated with ordinary NETs, especially those located in the periampullary region [7, 8]. 

It is unknown whether an association exists between NF1 and GCC. In the following we describe a patient with both diseases.

## 2. Patient

**Figure 1:**
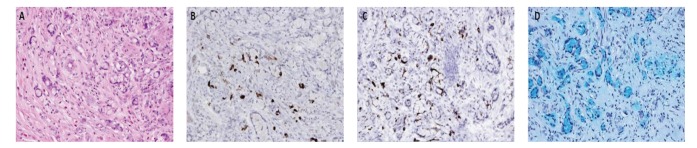
Histopathological examination. (A) Goblet cell carcinoid composed of cells with signet ring cells appearance with varied dilated cytoplasm due to mucin production withdisplacement ofthe nuclei to the periphery of the neuroendocrine cells location in the cytoplasm (HE, ×200); ((B) and (C)) positive staining for chromogranin A (B) and synaptophysin (C) demonstrating neuroendocrine differentiation (×200); (D) mucin stain (Alcian Blue, ×200).

A 60-year-old woman with known NF1 displaying the classical features including multiple neurofibromas was admitted with signs of acute appendicitis. She presented lower right-sided abdominal pain, distended abdomen, and elevated infection parameters and underwent laparoscopic appendectomy. She was in good health until the appendicitis and had never experienced any carcinoid symptoms. Besides an inflammatory affected appendix the pathology report showed a tumor with multiple infiltrating microglandular elements of goblet cells with signet ring cell features appearing as a goblet cell carcinoid subtype B (Figure 1(A)). The diagnosis of a goblet cell carcinoid was confirmed by positive immunostaining for the neuroendocrine markers chromogranin A (CgA) (Figure 1(B)) and synaptophysin (Figure 1(C)). Further, mucin was seen in Alcian blue-stained slides (Figure 1(D)). Proliferation index showed a Ki67 index of 30–40%. There was no sign of extension throughout the appendix wall but the resection margins were positive. Therefore, a right-sided hemicolectomy was performed with free margins and no lymph node metastases. Serologically, tumor markers serotonin and CgA were slightly increased while urinary 5-HIAA, carcinoembryonic antigen (CEA), cancer antigen 19-9 (CA 19-9), and cancer antigen 125 (CA 125) were all normal after surgery. Computer tomography (CT) scan showed no signs of metastases in the abdomen or thorax, which indicates the cancer has been radically resected. The somatostatin receptor scintigraphy was negative as expected [9]. 

## 3. Discussion

To the best of our knowledge this is the first report of a patient with GCC and NF1. The majority of GCC patients present in their fifth or sixth decades [10] with a slight female preponderance [11, 12]. Though the combination of NF1, and GCC could be coincidental both conditions are rare, and the expected chance of having both is approximately 0.00017 per million/year. As goblet cells derive of neuronal ectoderm, they are likely to express neurofibromin. Thereby, they may have an increased tumor risk with a biallelic haploinsufficiency of *NF1* gene. This would be in line with the association between NF1 and other NETs.

Usually, GCCs stain scattered positively for the neuroendocrine markers chromogranin A and synaptophysin but similar to the present case, they also produce mucin like colorectal adenocarcinomas [13]. Only a few GCCs have somatostatin receptors and somatostatin receptor scintigraphy for diagnosis, and staging is usually negative. The standard NET markers chromogranin A, serotonin, and U-5HIAA are usually normal or only slightly increased. Though present in more than 50% of patients with small intestinal NET [14], the carcinoid syndrome including diarrhoea and flushing is absent in patients with GCC. Followup after GCC is a clinical challenge and will usually include regular CT/MRI imaging combined with blood tests for CEA, CA 19-9, and CA 125. The latter may be elevated in some patients and serve as a tumor marker. If recurrence is suspected, FGD-PET may be of value [9].

Most GCCs are localized at the time of diagnosis and have a good prognosis with overall 5-year survival of 75% [10]. GCCs are significantly more malignant than typical NETs both of the appendix and in general [11, 15] with less than 20% median 5-year survival in disseminated disease [10]. 

This is the first case of GCC in an NF1 patient. The treatment of GCC in NF1 should not differ from other GCC patients as it includes appendectomy and right-sided hemicolectomy with lymph node dissection. GCC frequently metastasizes to the ovaries but routine hysteronsalpingo-oophorectomy is only recommended in the presence of ovarian metastases. In metastatic disease peritonectomy and intraoperative chemotherapy may be an option, and the use of chemotherapy follows the principles for treatment of colorectal adenocarcinoma [9, 16].

## References

[1] Yao JC, Hassan M, Phan A (2008). One hundred years after “carcinoid”: epidemiology of and prognostic factors for neuroendocrine tumors in 35,825 cases in the United States. *Journal of Clinical Oncology*.

[2] Modlin IM, Lye KD, Kidd M (2003). A 5-decade analysis of 13,715 carcinoid tumors. *Cancer*.

[3] Modlin IM, Kidd M, Latich I, Zikusoka MN, Shapiro MD (2005). Current status of gastrointestinal carcinoids. *Gastroenterology*.

[4] McGory ML, Maggard MA, Kang H, O’Connell JB, Ko CY (2005). Malignancies of the appendix: beyond case series reports. *Diseases of the Colon and Rectum*.

[5] Anderson NH, Somerville JE, Johnston CF, Hayes DM, Buchanan KD, Sloan JM (1991). Appendiceal goblet cell carcinoids: a clinicopathological and immunohistochemical study. *Histopathology*.

[6] Fuller CE, Williams GT (1991). Gastrointestinal manifestations of type 1 neurofibromatosis (von Recklinghausen’s disease). *Histopathology*.

[7] Ghidirim G, Rojnoveanu G, Mişin I, Cernîi A, Gurghiş R (2009). Carcinoid of the minor duodenal papilla associated with multiple jejunal leiomyomas in type 1 neurofibromatosis. *Chirurgia*.

[8] Hardt PD, Doppl WE, Klör HU, Hinrichs B (1998). A rare combination of a pheochromocytoma and a somatostatin-rich neuroendocrine tumor of Vater’s papilla in a patient with Recklinghausen’s neurofibromatosis. *Zeitschrift für Gastroenterologie*.

[9] Janson ET, Sørbye H, Welin S (2010). Nordic Guidelines 2010 for diagnosis and treatment of gastroenteropancreatic neuroendocrine tumours. *Acta Oncologica*.

[10] Roy P, Chetty R (2010). Goblet cell carcinoid tumors of the appendix: an overview. *World Journal of Gastrointestinal Oncology*.

[11] McCusker ME, Coté TR, Clegg LX, Sobin LH (2002). Primary malignant neoplasms of the appendix: a population-based study from the surveillance, epidemiology and end-results program, 1973–1998. *Cancer*.

[12] Tang LH, Shia J, Soslow RA (2008). Pathologic classification and clinical behavior of the spectrum of goblet cell carcinoid tumors of the appendix. *American Journal of Surgical Pathology*.

[13] Ramnani DM, Wistuba II, Behrens C, Gazdar AF, Sobin LH, Albores-Saavedra J (1999). K-ras and p53 mutations in the pathogenesis of classical and goblet cell carcinoids of the appendix. *Cancer*.

[14] Bergestuen DS, Aabakken L, Holm K, Vatn M, Thiis-Evensen E (2009). Small intestinal neuroendocrine tumors: prognostic factors and survival. *Scandinavian Journal of Gastroenterology*.

[15] Pham TH, Wolff B, Abraham SC, Drelichman E (2006). Surgical and chemotherapy treatment outcomes of goblet cell carcinoid: a tertiary cancer center experience. *Annals of Surgical Oncology*.

[16] Plöckinger U, Couvelard A, Falconi M (2007). Consensus guidelines for the management of patients with digestive neuroendocrine tumours: well-differentiated tumour/carcinoma of the appendix and goblet cell carcinoma. *Neuroendocrinology*.

